# Beyond Dizziness: Virtual Navigation, Spatial Anxiety and Hippocampal Volume in Bilateral Vestibulopathy

**DOI:** 10.3389/fnhum.2016.00139

**Published:** 2016-03-31

**Authors:** Olympia Kremmyda, Katharina Hüfner, Virginia L. Flanagin, Derek A. Hamilton, Jennifer Linn, Michael Strupp, Klaus Jahn, Thomas Brandt

**Affiliations:** ^1^German Center for Vertigo and Balance DisordersMunich, Germany; ^2^Department of Neurology, Ludwig-Maximilians UniversityMunich, Germany; ^3^Department of Psychiatry, Medical University InnsbruckInnsbruck, Austria; ^4^Department of Psychology, University of New MexicoAlbuquerque, NM, USA; ^5^Institute for Diagnostic and Interventional Neuroradiology, University Hospital Carl Gustav CarusDresden, Germany; ^6^Department of Acute Neurology, Schön Klinik Bad AiblingBad Aibling, Germany; ^7^Institute for Clinical Neurosciences, Ludwig-Maximilians UniversityMunich, Germany

**Keywords:** hippocampal atrophy, loss of vestibular function, navigation strategies, spatial anxiety, spatial orientation

## Abstract

Bilateral vestibulopathy (BVP) is defined as the impairment or loss of function of either the labyrinths or the eighth nerves. Patients with total BVP due to bilateral vestibular nerve section exhibit difficulties in spatial memory and navigation and show a loss of hippocampal volume. In clinical practice, most patients do not have a complete loss of function but rather an asymmetrical residual functioning of the vestibular system. The purpose of the current study was to investigate navigational ability and hippocampal atrophy in BVP patients with residual vestibular function. Fifteen patients with BVP and a group of age- and gender- matched healthy controls were examined. Self-reported questionnaires on spatial anxiety and wayfinding were used to assess the applied strategy of wayfinding and quality of life. Spatial memory and navigation were tested directly using a virtual Morris Water Maze Task. The hippocampal volume of these two groups was evaluated by voxel-based morphometry. In the patients, the questionnaire showed a higher spatial anxiety and the Morris Water Maze Task a delayed spatial learning performance. MRI revealed a significant decrease in the gray matter mid-hippocampal volume (Left: *p* = 0.006, *Z* = 4.58, Right: *p* < 0.001, *Z* = 3.63) and posterior parahippocampal volume (Right: *p* = 0.005, *Z* = 4.65, Left: *p* < 0.001, *Z* = 3.87) compared to those of healthy controls. In addition, a decrease in hippocampal formation volume correlated with a more dominant route-finding strategy. Our current findings demonstrate that even partial bilateral vestibular loss leads to anatomical and functional changes in the hippocampal formation and objective and subjective behavioral deficits.

## Introduction

Bilateralvestibulopathy (BVP) is characterized by impairment or complete loss of function of either the peripheral labyrinths or the eighth nerves (Baloh et al., 1989). The deficits can include both the semicircular canals and the otolith organs (Wiest et al., 2001). The most distressing symptom is unsteadiness of gait, which worsens in darkness and on uneven ground when vision and proprioception cannot substitute for the missing vestibular input (Crawford, 1952). About one half of patients also suffer from apparent motion of the visual scene (oscillopsia) during head movements or locomotion (Zingler et al., 2007), which is caused by involuntary retinal slip due to the deficient vestibulo-ocular reflex. With absence of motion, the patients are asymptomatic. BVP patients have a reduced quality of life with impaired social functioning (Guinand et al., 2012).

BVP is most commonly an adverse effect of aminoglycoside therapy or is caused by Meniere's disease or meningoencephalitis. However, in many patients, especially the elderly, the etiology remains unknown (Zingler et al., 2007). In the vast majority of these patients vestibular loss is incomplete and asymmetric. Electrophysiological testing, such as caloric irrigation and vestibular-evoked potentials, reveal that most patients have some residual vestibular function. For example, only 60% of the BVP patients with pathological canal function also had pathological otolith function (Agrawal et al., 2013). Total loss of peripheral vestibular function is very rare; it typically results from bilateral nerve surgical section in patients with Neurofibromatosis type II.

The human hippocampal formation (HF), i.e., the hippocampus (HC) and parahippocampus (PHC), is known to have functional importance in various aspects of memory (Scoville and Milner, 2000; Manns et al., 2003a,b) and to play an essential role in spatial memory and navigation (Maguire et al., 1996; Kessels et al., 2001; Spiers et al., 2001; Astur et al., 2002). Extensive navigational training (e.g., in taxi drivers) can lead to changes in HF volumes with smaller volumes in the anterior HF and larger volumes in the posterior HF (Maguire et al., 2000). Patients with vestibular nerve sections as well as those with extensive vestibular training display altered HF volumes (Brandt et al., 2005; Hufner et al., 2007, 2011a). Patients recovering from vestibular neuritis, which leads to an acute unilateral vestibular deficit, also show volume changes in the HF (zu Eulenburg et al., 2010).

In an earlier study in patients with bilateral vestibular nerve section we found deficits in spatial memory and navigation as well as hippocampal atrophy (Brandt et al., 2005). This suggests that a functioning vestibular system is important for spatial memory and navigation. A unilateral vestibular loss, however, did not greatly affect spatial memory or navigational performance, probably because sufficient information flows from the healthy side (Hufner et al., 2007). It remains to be seen if reduced and probably faulty input on both sides leads to such deficits, despite the same residual function.

In this study we assessed the navigational difficulties of patients with severe, but incomplete bilateral vestibulopathy, and possibly accompanying structural changes in the brain. In particular spatial memory and navigation performance were quantitatively evaluated with the virtual Morris water maze. Self-report questionnaires on spatial anxiety and spatial strategy were administered to investigate navigational problems in daily life. Finally, to examine structural changes in brain regions relating to navigation the hippocampal and parahippocampal gray matter volumes of patients and healthy controls were compared using voxel-based morphometry.

Our aim is to precisely characterize functional and structural changes in spatial memory and navigation in BVP in order to help physicians and physiotherapists recognize and treat these deficits.

## Materials and methods

### Patient characteristics

Fifteen patients with BVP (9 males, mean age 63.6 ± 11.5 years) with unsteadiness of gait were recruited from the German Centre for Vertigo and Balance Disorders, Munich. BVP was defined as (1) a bilateral pathological head-impulse test established by at least two experienced clinicians (O.K. and K.H.) and (2) a bilaterally reduced (mean peak slow phase velocity ≤6°/s) or absent responsiveness to bithermal (44 and 30°) caloric irrigation. Mean peak slow phase velocity (SPV) was calculated as the average of the peak slow velocities of the bithermal caloric-induced nystagmus on both sides. The cut-off of 6°/s refers to internal laboratory values, also in accordance with the literature (Vesterhauge and Kildegaard Larsen, 1977). Disease duration was 13.6 ± 17.4 years. Patients with clinical signs indicating dementia or mild cognitive impairment, cerebellar involvement or other neurological diseases were excluded from the study. Fifteen age- and sex-matched control subjects (CON) (mean age 63.6 ± 10.02 years), without a history of neurological diseases, vertigo or dizziness, and with a normal clinical head-impulse test and basic neurological clinical examination were also assessed. None of the subjects of the two groups were taking medication that directly affected cognitive function, e.g., antidepressants or antipsychotic drugs.

MRI scanning was not possible in two BVP patients and two CON due to contraindications for scanning. Handedness was determined according to the Edinburgh handedness inventory (Oldfield, 1971). Current clinical symptoms (only applicable to patients), including oscillopsia and ability to read signs while moving, as well as daily physical activities were assessed using a standardized questionnaire. The BVP and CON groups were also asked about the frequency of computer use (daily vs. 2–5 days/week vs. seldom/never). Questionnaire results were compared using Chi-Square (χ^2^) test.

The study was approved by the local ethics committee of the Ludwig-Maximilians University and conducted in accordance with the principles described in the Declaration of Helsinki. All subjects gave their written informed consent prior to the study.

### Spatial navigation and spatial anxiety

To quantify the patients' self-evaluation of their spatial performance two clinical scales were used: the Wayfinding Scale and the Spatial Anxiety Scale (Lawton, 1994). The Wayfinding Scale measures the degree to which participants use either a route (self-based position, Cronbach alpha = 0.73 for internal consistency) or an orientation strategy (environment-based, Cronbach alpha = 0.65) for navigation. The higher the value, the more the subjects employed the strategy being assessed. Persons with a high route strategy value tend to focus on specific instructions on how to get from place to place based on landmarks in the environment without a larger understanding of the environment (Lawton, 1994). A high score on the orientation strategy means that persons tend to develop a map-like or survey understanding of the environment and can update their own position within this environment (Lawton, 1994). Previous work showed that only a fraction of the population is able to develop this type of survey knowledge (Ishikawa and Montello, 2006), which is thought to depend on the hippocampus (Rodgers et al., 2012; Wiener et al., 2013). BVP patients were therefore expected to have a lower orientation score and higher route score, and the orientation strategy was expected to positively correlate with hippocampus gray matter volume, whereas the route strategy was expected to negatively correlate with the volume of the hippocampus.

The Spatial Anxiety Scale (Cronbach alpha = 0.80) is a 5-point Likert-type scale that assesses the anxiety participants have in eight daily life situations requiring spatial or navigational skills. The higher the value, the more strenuous the spatial tasks are for the subject. Both tests are indirect measures of spatial ability (Lawton, 1994). Results were weighted according to Lawton (1994), and group comparisons were performed using Student's *t*-test. The results of the Wayfinding scale and the Spatial Anxiety Scale were correlated using Pearson's correlation, according to Lawton (1994). *P* < 0.05 was considered significant for these analyses.

### Memory assessment

All tests were administered to all participants (BVP and CON). A German-language adaptation of the national adult reading test of Nelson was selected to estimate premorbid intelligence (MWI-B Test) (Lehrl, 2005). The following subtests of the Wechsler Memory Scale-Revised were administered to measure general memory performance (Härting et al., 2000): Mini Mental Test, Visual Reproduction I and II, Logical Memory I and II, Figural memory and Digit Span. Raw test values were transformed into percent ranges according to the age norms of the respective task (subjects >75 years were included in the eldest age group of 65–74). The Doors sub-test of the Doors and People Test (Baddeley et al., 1994) was used as a measure of long-term visual recognition memory. The normative results were compared using Student's *t*-test in all memory tests. *P* < 0.05 was considered significant for these analyses.

### Virtual morris water task (vMWT)

The Morris water task is considered the gold standard for testing spatial learning, spatial memory, and navigation in rodents (Morris, 1984). The virtual version of this test and its validation for determining human spatial learning, spatial memory, and navigation abilities are described in detail elsewhere (Hamilton et al., 2002; Driscoll et al., 2003). The test has been shown to effectively detect deficits in spatial memory in an elderly population (Driscoll et al., 2005) and in patients with bilateral vestibular failure (Brandt et al., 2005). Here a version of the virtual test adapted for elderly subjects was used: hidden and visible platform trials were applied before each testing phase. The subjects were given written instructions prior to testing, the testing procedures were explained in detail, and subjects had ample time to ask their questions of the investigator. A training phase with one or more test trials before the experimental trials allowed the subject to familiarize herself/himself with the required task. During the trial no assistance or verbal clues were given. Both groups received the same protocol.

**Figure 1:**
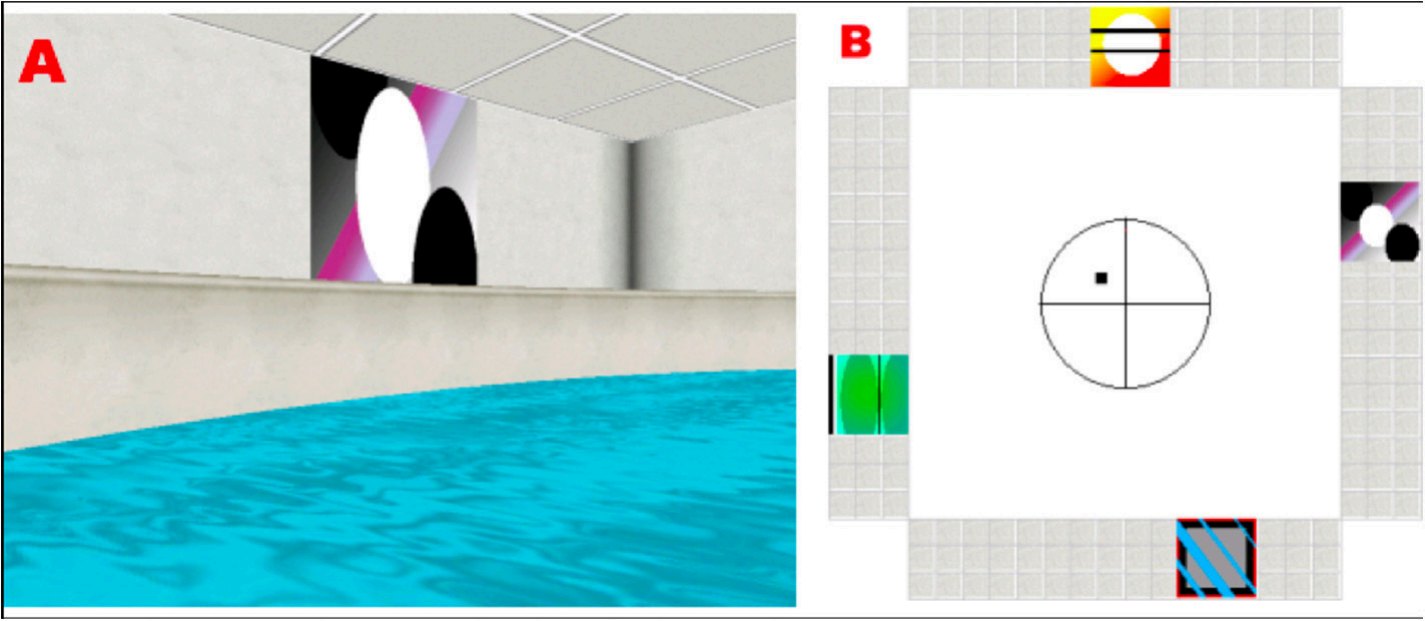
**(A)** Example of a visual cue as seen from the subject's perspective, while navigating in the platform. **(B)** Floor plan of the virtual room, indicating the position of the cues and the platform (black square).

In brief, the basic features of the environment consisted of a circular pool located in the center of a room with a square floor plan (Figure 1). Four conspicuous cues of equal size were placed around the distal walls. The cues were positioned so that one cue was on each of the four distal room walls, and the platform could not be encountered by simply moving toward a single cue from any release point. The platform was positioned in the center of one quadrant (N/E) and occupied 2% of the pool area. A first-person view of the virtual environment was displayed. The observer's position was always slightly above the surface of the water, and forward movement was controlled by the UP (:) arrow key on the keyboard. Rotation was controlled by the LEFT (/) and RIGHT (?) arrow keys. Backward navigation or up-down movement within the pool was not possible. A full 360° rotation in the absence of forward movement required 2.5 s to complete, and the direct path from a release point to the opposite side of the pool tool 4 s.

Three phase trials were performed:

1. Phase I consisted of 10 blocks with 2 visible platform trials each (i.e., a total of 20 trials)
2. Phase II consisted of finding a hidden platform that was always in the same position starting from different points and
3. Phase III consisted of a single, 45-s-long probe trial during which the platform was removed from the environment.

The exact duration of the whole task depended on how fast the subjects found the platform. The time limit for trials I and II was 60 s.

Three measures were computed for each training trial: latency and path length to the platform in each trial and the heading error (deviation from a direct trajectory to the platform measured when the subject had traveled 25% of the pool diameter from the release point in degrees). Group comparisons (BVP and CON) were conducted with separate repeated measures analyses of covariance (RM ANCOVAs) with sex and group as between-subjects factors, trial block as a within-subjects factor and age as a covariate. To provide an additional qualitative assessment for hidden platform training trials three raters blind to group membership classified each participant as either (1) learning to execute direct trajectories to the platform from each release point or (2) using a strategy that did not result in execution of direct trajectories. These values were compared between BVP patients and CON using separate, repeated measures ANCOVA analyses of covariance with sex as between-subjects factors and with trial block as a within-subjects factor, and age as a covariate.

For the probe trial (Phase III) the time and distance spent in the platform quadrant were additionally assessed. A multivariate ANCOVA (MANCOVA) was performed, since the measures are all inherently correlated, with percent time and path length in the platform quadrant, latency and path length to enter the platform quadrant as dependent measures, and subject group and sex as between-subjects factors with age as a covariate.

Data of one BVP patient and one control could not be analyzed for technical reasons.

There were significant negative relationships between age and all dependent measures reported here (*p* < 0.001); therefore, age was included as a covariate for all analyses of the data from the vMWT.

### Brain MRI analysis

Acquisition protocol: Acquisitions were done with a 3-T GE Signa HDx scanner using an eight-channel head coil. A 3-D gradient-echo sequence (FSPGR fast spoiled gradient recalled), with a voxel size of 0.86 × 0.86 × 1.4 mm with 0.7 mm oversampling in the z direction was used to acquire T1-weighted brain images from all subjects (TE: 3.2 ms, TR: 7.9 ms, Ti: 500 ms, flip angle: 15°).

#### Voxel-based morphometry (VBM)

VBM was used to analyze the 3-D FSPGR data sets. Data were processed using SPM8 (Wellcome Department of Cognitive Neurology, London, UK, http://www.fil.ion.ucl.ac.uk/spm) with the integrated VBM toolbox (VBM8, http://dbm.neuro.uni-jena.de/vbm/) and Matlab (MathWorks, Natick, MA, USA). Data sets of left-handed subjects (75–100% left handedness on the Edinburgh handedness inventory (Oldfield, 1971): three in the BVP group and one in the CON group) were flipped and then analyzed together with right-handed data sets. This step was applied since a lateralization of the cortical representation (right hemisphere dominance for right-handers and left hemisphere for left-handers) of the vestibular system was described previously (Dieterich et al., 2003). The following preprocessing steps of the VBM toolbox were applied: spatial normalization to the space defined by the MNI template, tissue classification, and registration using linear and non-linear transformations (warping) within the same generative model (Ashburner and Friston, 2005). Analysis was performed on modulated gray matter (GM) segments. They were multiplied by the non-linear components derived from the normalization matrix to preserve actual GM volumes locally, and then smoothed with an 8-mm isotropic Gaussian kernel. The confounding effects of individual differences in brain orientation, alignment, and different brain sizes were accounted for by applying modulation for non-linear warping only (Luders et al., 2009).

Voxel-wise GM differences between BVP patients and controls were compared using statistical parametric mapping software (SPM8) and a general linear model with one dependent variable (the voxel-wise GM volume changes) and multiple independent variables (univariate multiple linear regression, where all of the factors were considered simultaneously). The following independent variables were included in the analysis: results of the Doors Test, Wayfinding Score Route Strategy and Orientation Strategy, premorbid intelligence (MWT-B), Spatial Anxiety Scores, vMWT data “percent time in target quadrant” and “latency to target quadrant,” gender and age. Care was taken to select the independent variables, so as to exclude their correlation. Parameters were estimated using a least-squares approach; the t-statistic is a valid statistic for testing whether the slope of the relationship between the dependent variable and a linear combination of the independent variables is greater than zero. Grubb's test was used to identify potential outliers. Only clusters of 20 voxels or more were included in the analysis. Voxel-wise statistical parametric maps were created, which identified brain regions containing significant differences of local GM volume for the contrasts of interest; familywise error correction (FWE) was applied (Wright et al., 1995; Ashburner and Friston, 1997). A region of interest (ROI) was created for the hippocampus (HC), the parahippocampus (PHC), grouped together as the hippocampal formation (HF) and the caudate nucleus using the WFU-Pickatlas (ANSIR, Wake Forest University; Maldjian et al., 2003). Anatomical structures were named according to the Automated Anatomical Labeling Atlas (Tzourio-Mazoyer et al., 2002). The results for the hippocampus were described as located in the “anterior,” “middle,” or “posterior” hippocampus depending on the y coordinates obtained, since different hippocampal regions are implicated in distinct cognitive processes (Fanselow and Dong, 2010; Hufner et al., 2011a). This distinction was made by dividing the hippocampus into three regions along the Y-axis as described by Greicius et al. (2003). The MNI space was used in the current study.

## Results

### Patient characteristics

The clinical characteristics and findings for the BVP patients are given in Table 1. All patients hat a clinically pathological head-impulse test (Halmagyi and Curthoys, 1988). BVP and CON did not differ in frequency of computer use and daily level of physical activity (Chi-Square, *p* > 0.05).

**Table 1:** **Clinical data of patients with bilateral vestibulopathy**.

**Patient**	**Age (y)**	**Sex**	**Disease duration (y)**	**Etiology**	**Mean SPV (°/s)**	**Oscillopsia**	**Other diseases**	**Hobbies**
BVP1	79	m	10	Aminoglycosides	0.9	No	Atrial fibrillation, hypertension	None reported
BVP2	86	f	69	Inflammatory	0	Yes	Hearing loss, atrial fibrillation, diabetes	None reported
BVP3	58	m	8	Idiopathic	1.5	Yes	Tinnitus	Nordic walking
BVP4	58	m	5	Idiopathic	0	Yes	Dyslipidemia	Cycling, skating
BVP5	67	f	15	Inflammatory	1.2	Yes	Chronic back pain, hypertension	Hiking
BVP6	58	m	15	Traumatic	4.5	Yes	Chronic back pain	Computer games
BVP7	68	f	12	Inflammatory	1.6	Yes	Sjögren's syndrome	Self-help group leader
BVP8	65	m	35	Inflammatory	0	Yes	None	Computer games
BVP9	63	m	2	Idiopathic	1.8	No	Hypertension	Cycling, skiing, nordic walking
BVP10	44	f	10	Idiopathic	0	No	Hearing loss	Skiing, swimming
BVP11	45	m	4	Idiopathic	1	No	Asthma	Cycling
BVP12	61	m	2	Idiopathic	0	Yes	Prostate hyperplasia	Swimming, reading
BVP13	66	f	12	Menière's disease	6	No	Hypertension	Reading
BVP14	59	f	2	Idiopathic	5.3	No	Diabetes	Horse back riding
BVP15	78	m	4	Idiopathic	2	No	Hypertension	Cycling, ping pong

*SPV, Slow phase velocity; HIT, head impulse test (Halmagyi and Curthoys, 1988)*.

### Neuropsychological assessment

All patients and normal subjects completed all cognitive tasks and questionnaires (except for BVP 1 who did not complete the MWI-B Test and BVP 10 that left out a part of the Wayfinding scale). Subjects of the BVP group had significantly higher scores in the Spatial Anxiety Scale compared to controls [*t*-test, *t*_(28)_ = 2.4, *p* = 0.023, *r* = 0.46; Table 2]. No significant group differences were observed in navigational strategies (orientation vs. route strategy) as measured by the Wayfinding Scale [Orientation strategy: *t*_(27)_ = 1.7, *p* = 0.25, *t*_(27)_ = 1.7, *p* = 0.15, Table 2]. No significant correlation between scores on the Wayfinding Scale and Spatial Anxiety Score was observed (Pearson correlation, *p* > 0.05). No differences in intelligence as measured by the reading test [MWI-B Test, *t*_(27)_ = −0, 0.170, *p* = 0.87], in visual recognition memory as measured by the Doors test [*t*_(28)_ = −0.43, *p* = 0.67] or in general memory as evaluated by the Wechsler Memory Scale Revised, [Mini Mental Test (*t*_(28)_ = −0.46, *p* = 0.65), Visual Reproduction I (*t*_(28)_ = −1.9, *p* = 0.7) and II (*t*_(28)_ = −0.7, *p* = 0.49), Logical Memory I (*t*_(28)_ = 0.1, *p* = 0.92) and II (*t*_(28)_ = −1.05, *p* = 0.3), Figural memory (*t*_(28)_ = −0.41, *p* = 0.69) and Digit Span (*t*_(28)_ = 0.2, *p* = 0.67) were detected between groups].

**Table 2:** **Results of Wayfinding Scale (orientation and route strategies) and Spatial Anxiety Scale**.

**Group**		**Orientation strategy**	**Route strategy**	**Spatial anxiety**
BVP	Mean	13.86	8.49	14.96^*^
	Std.	3.17	4.08	5.13
CON	Mean	15.53	10.54	11.00^*^
	Std.	4.33	3.42	3.77
	*p*-value	0.246	0.156	**0.023**

*Higher values indicate increased use of the respective navigational strategy or increased prevalence of spatial anxiety. The asterisk indicates statistical significance between the BVP and CPN group. For exact statistical values see text. Bold indicates p value below 0.05*.

### Spatial memory and navigation

#### Visible platform navigation (phase I)

No significant effects or interactions involving the group and trial block factors on latency, path length or heading error were found (ANCOVA for latency, path, length and heading error, *p* > 0.10 in all tests). Males were significantly faster than females [latency in males < females; *F*_(1, 23)_ = 8.108, *p* = 0.009], and more precise [heading error males < females; *F*_(1, 23)_ = 11.142, *p* = 0.003, *r*^2^ = 0.326], as shown in previous studies (Hufner et al., 2007); however, there were no other significant main effects or interactions involving gender (*p* > 0.08; Data not shown).

#### Hidden platform navigation (phase II)

**Figure 2:**
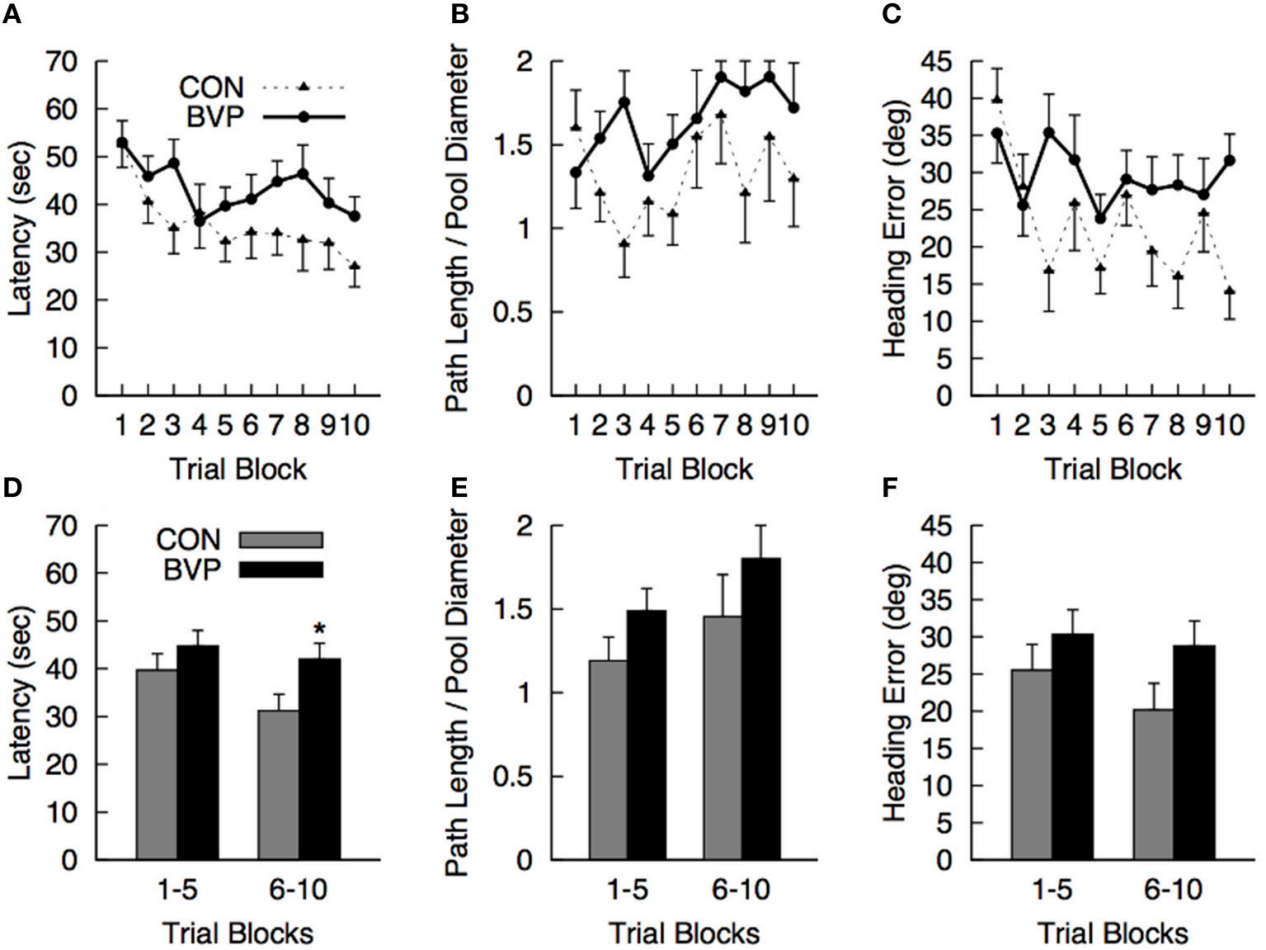
**Mean (+SEM) measures during the hidden platform training (Phase II) for the control (CON) and bilateral vestibulopathy (BVP) groups; (A) Latency to navigate to the platform, (B) Path length to the platform, expressed as Path Length/Pool Diameter, (C) Heading error**. (D–F)represent the same measures averaged over the first 5 and last 5 training blocks. ^*^significant group effect at *p* < 0.05.

No significant effects or interactions involving the factors of interest were detected (ANCOVA; Figures 2A–C). Comparison of mean values for each dependent measure during the first 5 (1–5) and last 5 (6–10) trial blocks revealed that controls were finding the platform faster than the patients during the last 5 blocks of training [latency BVP > CTR, *F*_(1, 23)_ = 4.46, *p* = 0.046, *r*^2^ = 0.163] [Figures 2D–F). All other effects and interactions were not significant (*p* > 0.05)].

#### Probe trial (phase III)

**Figure 3:**
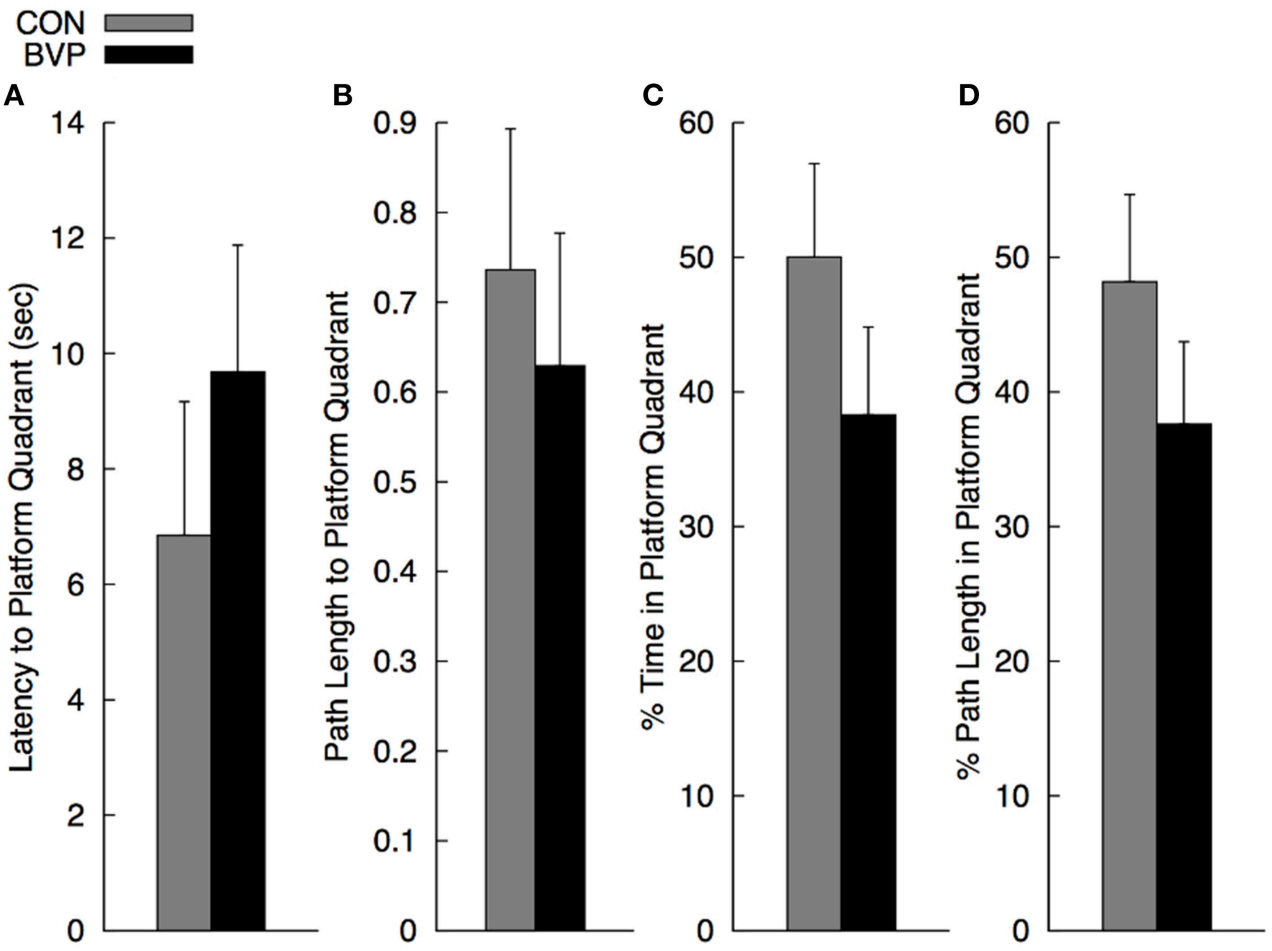
**Mean (+SEM) measures during the no-platform probe trial (Phase III) for the control (CON) and bilateral vestibulopathy (BVP) groups; (A) Latency to enter the platform quadrant, (B) Path length to the platform quadrant, expressed as Path Length/Pool Diameter, (C) Percent time spent in the platform quadrant, and (D) Percent path length spent in the platform quadrant**.

As mentioned before, multiple ANCOVA was used for statistical analysis. Results are shown in Figure 3. There was a significant multivariate effect of group [Wilks' Λ: 0.485; *F*_(4, 20)_ = 5.30, *p* = 0.004]. The main effect of gender and of group × gender interaction was not significant (*p* > 0.10). The standardized discriminate function coefficients for the multivariate group effect were ordered as follows: percent path length to platform quadrant (3.06), latency to enter the platform quadrant (−2.67), percent path length in the platform quadrant (1.48) and percent time in the platform quadrant (−0.56), indicating that the groups were best discriminated by variables related to navigating quickly and directly to the platform quadrant. Variables related to persistence in searching were somewhat less discriminating. It is important to note that the greater path length to navigate to the platform quadrant in controls compared to BVP patients was largely due to the two significant outliers (>2 standard deviations). When removed, the mean path length for group CON dropped significantly (26%) to 0.55 (SEM = 0.9). However, because of the high variability in the individual measures, none of the direct comparisons among groups for the individual dependent measures were significant (*p* > 0.30).

### MRI analysis

#### Voxel-based morphometry of gray matter volumes

**Figure 4:**
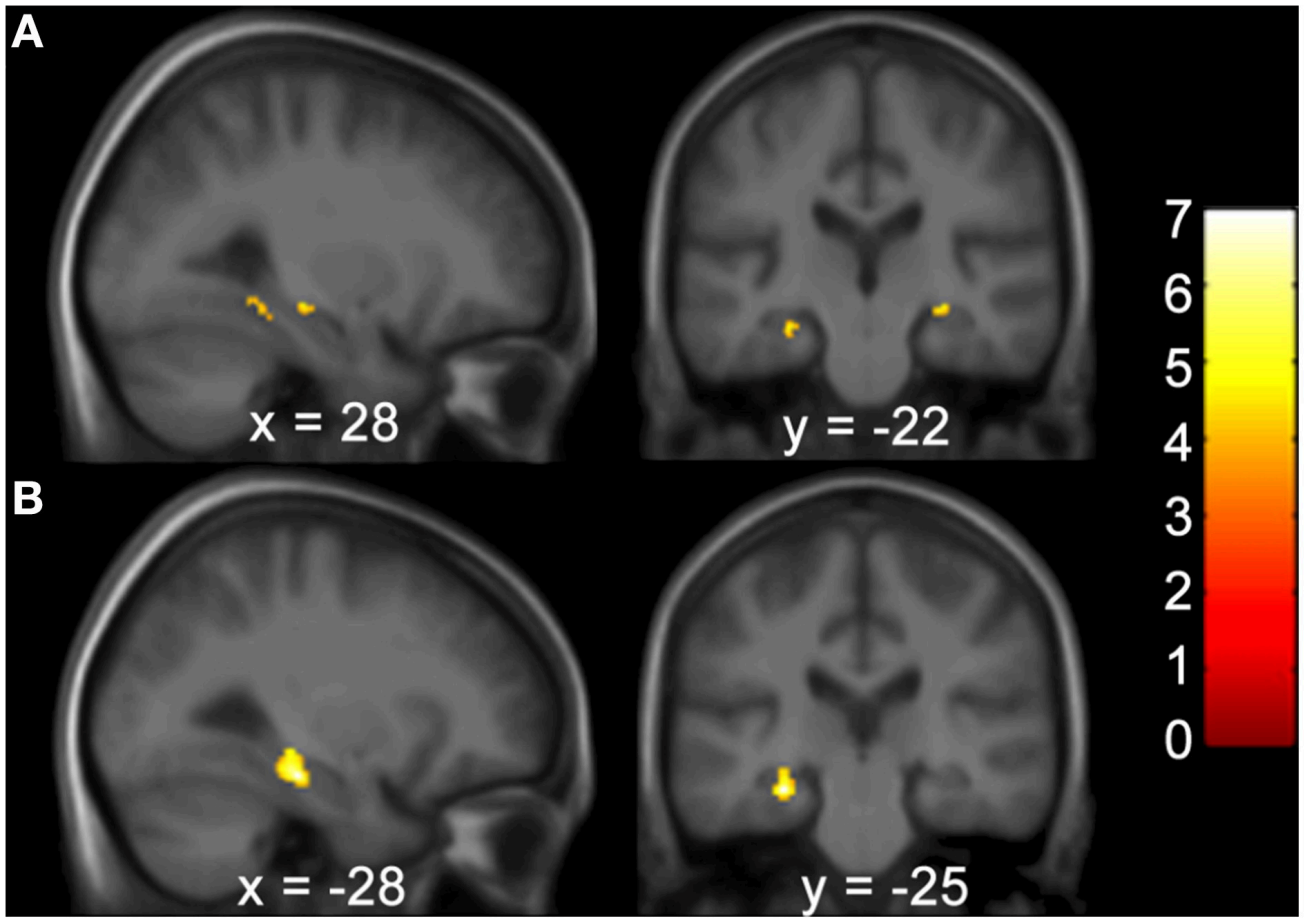
**Areas of higher GM volume in healthy controls compared to BVP patients. (A)** Clusters of GM differences between the two groups are shown on sagittal and coronal planes through the maximum cluster in the right hemisphere at 28 −22 −9 at *p* < 0.001 uncorr. and **(B)** left hemisphere at −28 −25 −17 *p* < 0.05 FWE corr. Clusters and significance values are from an ROI analysis of the HC and PHC bilaterally and are projected onto a mean image of the included subjects. Color bars indicate the range of *t*-values. The threshold for statistical significance was 3.69.

Gray matter brain volumes of BVP patients were compared to those of matched controls using voxel-based morphometry (Wright et al., 1995; Ashburner and Friston, 1997; Critchley et al., 2003). Whole brain analysis did not reveal any areas of gray matter change (*p* > 0.05, FWE corr.) for both contrasts (BVP > CON and CON>BVP). A ROI analysis of the HC and PHC detected no gray matter changes for the contrast BVP > CON (*p* > 0.05 FWE corr. or 0.001 uncorr.). For the contrast CON > BVP loss of gray matter was found in the HC and PHC bilaterally (Table 3, Figure 4). This included the mid-hippocampus bilaterally with a maximum at *y* = −22 right and at *y* = −25 left, reaching into the posterior HC on the left side, as well as the posterior PHC bilaterally (*y* = −37). No differences were observed in the caudate nucleus between the two groups.

**Table 3:** **GM coordinates and cluster sizes of areas of the hippocampal formation with larger GM volume in healthy controls compared to BVP patients**.

***P*-value FWE corr**.	***T***	***Z***	**Cluster size**	**Coordinates**	**Label**
**CONTRAST: COP**>**BVP PATIENTS**					
0.005	6.95	4.65	211	22 −37 −12	R PHC
0.006	6.75	4.58	155	−28 −25 −17	L HC
0.000^*^	5.09	3.87	20	−20 −37 −12	L PHC
0.000^*^	4.63	3.63	28	28 −22 −9	R HC

*The asterisk indicates a threshold of p < 0.001 uncorrected for multiple comparisons. Statistical values are from ROI analysis including the hippocampus and parahippocampus. ROI, Region of Interest; GM, Gray Matter; R, Right; L, Left; PHC, Parahippocampus; HC, Hippocampus*.

#### Relationship between HF volume and spatial performance

**Figure 5:**
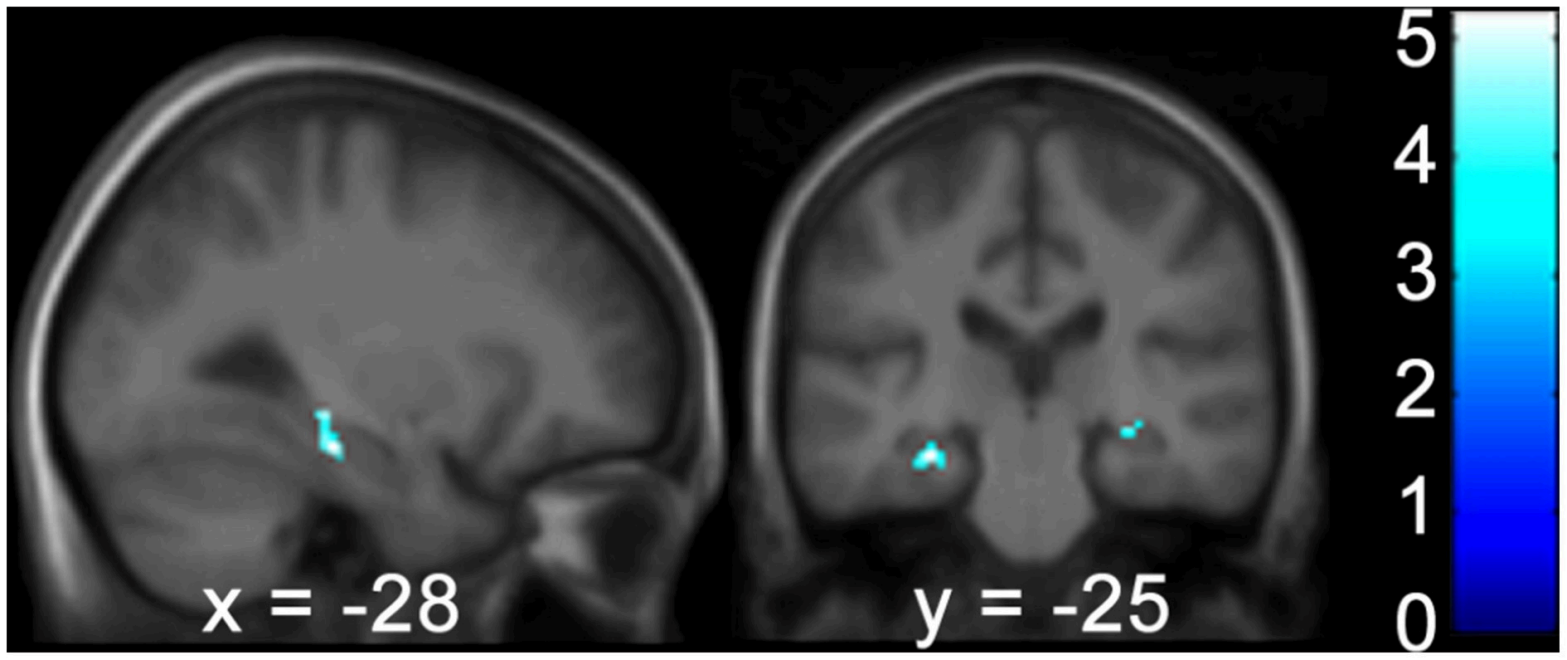
**The Wayfinding Scale route strategy scores correlate negatively with GM hippocampal volume bilaterally**. Results are shown in a sagittal and coronal plane through the peak voxel at −28 −25 −17 and with the PHC GM volume on the right (not visible here). (*p* < 0.001 uncorr., ROI analysis of the HC and PHC bilaterally). Color bars indicate the range of *t*-values. The threshold for statistical significance was 3.69. PHC, Parahippocampus; GM, Gray Matter; ROI, Region of Interest; HC, Hippocampus.

Correlation analyses were performed to test for an effect of the covariates on regional GM brain volumes in the HF and caudate nucleus in patients and controls, separately and grouped together. For the results of the Doors Test, Wayfinding Score orientation strategy, premorbid intelligence (MWT-B), vMWT data “percent time in target quadrant” and “latency to target quadrant” and gender no significant effect was found (*p* > 0.001 uncorrected). However, results of the Wayfinding Score route strategy showed an inverse effect on HF volume: a maximum occurred at −28 −25 −17 (left middle HC reaching into the posterior HC), at 28 −24 −9 (right middle HC reaching into posterior HC), and at 22 −36 −11 (right posterior PHC) for the entire cohort (Table 4; Figure 5). The caudate nucleus data showed a positive correlation with the route strategy only at −6 10 −0 (*T* = 3.77, Z = 3.17, *p* = 0.001).

**Table 4:** **GM coordinates and cluster sizes of areas that correlate negatively with the route strategy score of the Wayfinding scale**.

***P* (uncorr)**	***T***	***Z***	**Cluster size**	**Coordinates**	**Label**
**WAYFINDING SCALE ROUTE STRATEGY**					
0.000	−5.21	−3.93	68	−28 −25 −17	L HC
0.000	−4.84	−3.74	44	28 −24 −9	R HC
0.000	−4.70	−3.67	75	22 −36 −11	R PHC

*Statistical values are from ROI analysis including the hippocampus and parahippocampus. ROI, Region of Interest; GM, Gray Matter; R, Right; L, Left; PHC, Parahippocampus; HC, Hippocampus*.

## Discussion

In the present study we demonstrate subtle deficits in spatial memory and navigation as well as atrophy of the mid-hippocampus and the posterior parahippocampus and increased spatial anxiety, all of which were detected in an unselected patient population with severe but incomplete BVP. The patients did not differ from controls in general memory or whole brain gray matter volume. The specific impairment of spatial memory and navigation detected in these patients together with the increased spatial anxiety contributes to a reduced quality of life as described earlier (Guinand et al., 2012; Agrawal et al., 2013).

### Increased spatial anxiety in patients with BVP

The Spatial Anxiety Scale was developed to quantify anxiety about environmental navigation (Lawton, 1994). This scale provides an objective measure of the difficulties that these patients face and takes into account daily situations requiring spatial navigation abilities, such as finding one's car in a parking lot or finding one's way in a supermarket. Patients showed on average higher scores than their matched controls. This result provides evidence that the spatial deficits in BVP, as shown in the current and previous studies (Brandt et al., 2005; Hufner et al., 2007) are not limited to laboratory test conditions. These deficits have been neglected so far in the clinical routine, partly because they are not captured by the standard Dizziness Handicap Inventory (DHI) score (Jacobson and Newman, 1990; Guinand et al., 2012). They are, however, clinically relevant since increased levels of anxiety can lead to an increase in secondary somatoform vertigo (Tschan et al., 2011) and further reduce the quality of life.

### Spatial memory and navigation are impaired in BVP

The increased spatial anxiety in BVP was associated with an objectively impaired spatial memory and navigation (measured by the virtual version of the Morris water task). The patients' impairment was most evident in the parameters measuring direct and rapid navigation to the platform quadrant in the probe trial, particularly during the late phase of learning. Furthermore, patients required more time for the less direct trajectories to the platform during the training phase than controls. These impairments were not as pronounced as those observed in patients with complete bilateral vestibular deafferentation (Brandt et al., 2005), most likely due to our patients' residual vestibular input. Such a pattern in the vMWT suggests that the degree of vestibular damage and the degree of impairment in place learning and memory are related. Mild impairments indicated in the vWMT were accordingly detected in patients with only right vestibular lesions (Hufner et al., 2007).

Impairments of performance in the vMWT have been reported for elderly subjects. They were particularly evident in subjects within the age ranges used in the present study (Driscoll et al., 2005). However, all patients and controls sampled here were matched for age. Because there were significant negative relationships between age and all dependent measures reported, age was included as a covariate for all analyses. Therefore, the observed impairments in spatial learning memory in the VMWT related to vestibular input represent effects above and beyond those attributable to normal age-related decline.

### BVP leads to atrophy of the hippocampal formation

It is well recognized that the vestibular system is relevant for human spatial memory and navigation as well as for the integrity of the hippocampal formation (HF), i.e., the parahippocampal region and the hippocampus proper. Several studies in humans have shown that there is an intimate interaction between the vestibular system and the HF, for example, the use of vestibular stimulation provided evidence of HF activation with functional imaging (Vitte et al., 1996; Suzuki et al., 2001; Dieterich et al., 2003) as well as neuropsychological changes in spatial memory (Bachtold et al., 2001). Human lesion studies showed that the abolition of vestibular input causes significant deficits in spatial memory as well as hippocampal volume changes (Schautzer et al., 2003; Brandt et al., 2005; zu Eulenburg et al., 2010; Alessandrini et al., 2013).

The present VBM analysis showed atrophy of the mid-hippocampus and the posterior parahippocampus bilaterally in patients with chronic, bilaterally reduced vestibular function. On the other hand, neither generalized brain atrophy nor atrophy of the caudate nucleus was noted. Patients with unilateral vestibular deafferentation after acoustic neurinoma removal did not show significant atrophy of the HF, probably because the unilateral vestibular input was preserved in the HF (Hufner et al., 2007). An fMRI study of subjects with bilateral vestibular deafferentation and blind subjects proposed that vestibular input enters the hippocampal formation at its anterior aspect, i.e., the entorhinal cortex, while the processing of visual information takes place in the more posterior aspects (Jahn et al., 2009). A meta-analysis supports this view of a spatial visual-vestibular separation of information processing in the hippocampal formation (Hufner et al., 2011b). In our meta-analysis the maxima of vestibular signals were located in the anterior and middle HC.

The maximum of HC atrophy was also located within this range but extended slightly more posteriorly on the left side (maxima at *y* = −25 left and −22 right). This is similar to the region (*y* = −23) found in a recent study on patients after unilateral vestibular neuritis; atrophy was only found on the left side of the HC irrespective of the side of vestibular failure (zu Eulenburg et al., 2010).

In the PHC, vestibular-related information was likely to be located more anteriorly and visual information more posteriorly, although this separation was less evident than in the HC (Hufner et al., 2011b). In the current analysis the volume changes in the PHC were located in the posterior aspects bilaterally, which, according to the model described, cannot directly be attributed to the loss of vestibular input. This suggests that a complex network for multisensory information processing is present in the HF.

### Measures of hippocampal volume and spatial performance

The relevance of the HF, the right side in particular, for spatial orientation and navigation has been revealed in both animal and human studies (Moser et al., 1993; Ghaem et al., 1997; Maguire et al., 1997; Gron et al., 2000; Astur et al., 2002; Hartley et al., 2003; Zhang et al., 2004). The patients in the present study did not differ from controls in their self-reported navigation strategies (route vs. orientation strategy), both of which were measured by the Wayfinding Scale; however, their performance in the vMWT was reduced. The GM volume of the HF did not correlate with any quantitative performance scores. Thus, there is no proof of a direct relationship between disease-related hippocampal volume changes and spatial navigation deficits. The only correlation between performance and GM volume was found for the middle-to-posterior hippocampus, including the right posterior parahippocampus. This volume also correlated negatively with the route strategy in our entire subject cohort. Therefore, with smaller the hippocampal size, the route strategy became more dominant.

The Wayfinding Scale consists of a set of questions weighted according to two different navigation strategies: orientation and route. The orientation strategy can be thought of as monitoring self-position information rather than external environmental cues (Lawton, 1994). Questions that are weighted toward this strategy include, but are not limited to, orientation or place strategies and the development of a cognitive map, which are known to be dependent on the hippocampus (Tolman, 1948; Cheung et al., 2012), particularly the posterior hippocampus (Janzen and van Turennout, 2004). It is strange that the orientation strategy showed no positive correlation with the hippocampus. However, previous studies showed that values from the route strategy are more sensitive to differences of gender (Lawton, 1994) and culture (Lawton and Kallai, 2002). Questions that are weighted toward the route strategy primarily rely on directions (Lawton, 1994), e.g., turn right at the next intersection. The elderly (Rodgers et al., 2012; Wiener et al., 2013) and women (Lawton, 1994) prefer such route-, response-based strategies. The negative correlation between route-based navigation and posterior hippocampal volume could reflect this higher sensitivity.

On the other hand, caudate nucleus showed only a weak correlation at −6 10 −0 with the route strategy, which on the whole is consistent with navigation strategies that do not depend on environmental clues (Bohbot et al., 2007). Nevertheless, it is difficult to draw any further conclusions based on this result.

Although the orientation strategy may be based more on self-position monitoring, both strategies have sensorimotor components that require accurate vestibular information for successful navigation. Indeed this may partially explain why no behavioral differences were found between our groups. However, it is still possible that given lower between-subject variability and a larger sample size, differences in behavior and the resulting reduction in hippocampal volume may become apparent.

### Effects of stress on hippocampal volumes

In light of the increased spatial anxiety scores of BVP patients, it is of interest that deficits of spatial memory and navigation have also been reported to occur in children with anxiety disorders (Mueller et al., 2009). Furthermore, adults with social phobias show higher cerebral blood flow in the anterior and middle hippocampal regions (*y* = −13) when anticipating speaking in public (Tillfors et al., 2002); this area includes the region showing GM atrophy in our patients. These findings point to a two-way interaction between anxiety and hippocampal volume which involves spatial memory and navigation. Fanselow and Dong (2010) proposed that the dorsal HC is involved in information processing (spatial orientation predominantly on the right side), and the ventral HC correlates with emotion and stress. This theory derived from evaluation of the expression of genetic markers as well as functional lesion studies. Their theory also supports the notion of an interaction between spatial memory and anxiety within the hippocampus.

A stress effect could be responsible for the discrepancies between human and animal studies. Animal studies have not so far shown hippocampal atrophy after bilateral vestibular damage (Besnard et al., 2012; Zheng et al., 2012). One contributing factor that should be taken into consideration (besides loss of vestibular input) is the higher anxiety levels described in patients with BVP (Guinand et al., 2012; Saman et al., 2012), partly due to social difficulties (Agrawal et al., 2013). Laboratory animals, on the other hand, are rather hyperactive after labyrinthectomy, which may help maintain hippocampal volumes (Zheng et al., 2012) and contrary to humans (Horii et al., 2007), they do not show higher levels of circulating corticosteroids after vestibular deafferentation (Lindsay et al., 2005; Russell et al., 2006).

## Conclusions

Our current findings demonstrate that partial bilateral vestibular loss also leads to anatomical and functional changes in the hippocampal formation, which are reflected in subjective and objective behavioral deficits. These deficits should be directly addressed by the attending physicians, when evaluating daily life challenges in those patients.

## Author contributions

OK designed the experiment, recruited subjects, acquired, analyzed and interpreted the data and drafted the manuscript. KH designed the experiment, acquired, analyzed and interpreted the data and drafted the manuscript. VF designed the experiment, acquired analyzed and interpreted the data and drafted part or the manuscript. DH designed the experiment, analyzed and interpreted the data and drafted part of the manuscript. JL substantially contributed to the conception of the experiment and acquisition and interpretation of the data and critically revised the manuscript. MS substantially contributed to the conception of the experiment, interpretation of the data and critically revised the manuscript. KJ substantially contributed to the conception of the experiment, interpretation of the data and critically revised the manuscript. TB substantially contributed to the conception and design of the experiment, interpretation of the data and critically revised the manuscript. All authors gave final approval for publication and agreed to be accountable for all aspects of the work in ensuring that questions related to the accuracy or integrity of any part of the work are appropriately investigated and resolved.

### Conflict of interest statement

The authors declare that the research was conducted in the absence of any commercial or financial relationships that could be construed as a potential conflict of interest.

## Abbreviations

ANOVA: analysis of variance
BVP: bilateral vestibulopathy patients
CON: control patients
GM: gray matter
HC: hippocampus
HF: hippocampal formation
PHC: parahippocampus
ROI: region of interest
vMWT: virtual Morris water task.
